# Mitigating the shadow economy through financial sector development in Indonesia: some empirical results

**DOI:** 10.1016/j.heliyon.2021.e08633

**Published:** 2021-12-18

**Authors:** Sugiharso Safuan, Muzafar Shah Habibullah, Eric Alexander Sugandi

**Affiliations:** a Faculty of Economics and Business, Universitas Indonesia, Indonesia; b Putra Business School, Malaysia; c Asian Development Bank Institute, Japan

**Keywords:** Shadow economy, Modified-cash-deposit-ratio, Financial sector development, Indonesia

## Abstract

We examine the relationship between financial sector development and the shadow economy in Indonesia from 1980 to 2020. We estimate the size of Indonesia's shadow economy using the “Modified Cash to Deposits Ratio” approach. We then construct a long-term model using the size of Indonesia's shadow economy as the dependent variable. We set financial sector development as the main independent variable in our model. We use per capita real gross domestic product, the misery index, and foreign direct investment as control variables in our model. We find that financial sector development and the size of Indonesia's shadow economy have a nonlinear relationship that shows an inverted U-shape curve. The size of the shadow economy expands at the early stages of financial sector development to a turning point and decreases when financial sector development increases further. We also find that foreign direct investment curtails Indonesia's shadow economy. Additionally, increases in income expand Indonesia's shadow economy while misery index shows ambiguous results. We suggest the Indonesian authorities widen access for micro, small, and medium firms to the credit markets and enhance existing programs to reduce poverty and narrow the income gap in the country. These efforts help to narrow the size of Indonesia's shadow economy.

## 1 Introduction

Many empirical economic studies have explored the relations between financial sector development and economic growth. Some studies find that financial sector development can boost and stimulate economic growth (Levine, 1997; Levine et al., 2000). Ram (1999) finds a weak negative impact of financial sector development on economic growth in low- and medium-growth countries but a strong positive impact on the high-growth countries. Deidda and Fattouh (2002) find that financial sector development has no significant impact on low-income countries but has a strong and positive impact on high-income countries. Dawson (2003) finds that financial sector development does not have a significant impact on economic growth in the central and eastern European countries.

Some economists suggest that financial sector development and economic growth may exhibit a non-linear and non-monotonic relationship, such as an inverted U-shaped curve (Law and Singh, 2014). Ergungor (2008) argues the heterogeneity in the financial structure will lead to a non-linear relationship between economic growth and financial sector development.

In brief, the effectiveness of financial sector development in stimulating economic growth is still debatable. Some studies on developing countries find that financial sector development can accelerate economic growth. The “supply leading” or the “financial-led growth” hypotheses posited by Patrick (1966), McKinnon (1973), and Shaw (1973) has helped developing countries to transform their economies from “financially repressed” economies to “more financially liberalized” economies that spur economic growth (World Bank, 1989). Studies by Habibullah and Eng (2006a) and Eng and Habibullah (2011) find that financial sector development spurs economic growth in some African, Asian, European, and Western Hemisphere countries.

**Figure 1:**
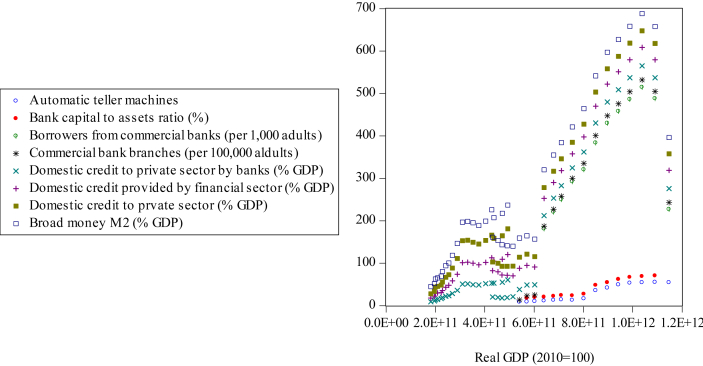
Scatter plots between real GDP and financial sector development indicators.

In the case of Indonesia, financial repression is far away from the past. Indonesia began its financial sector liberalization in 1978 when Bank Indonesia (BI) allowed time deposit rates to follow the market mechanism. BI eliminated restrictions on capital flows in the early 1980s (Habibullah and Smith, 1997). Figure 1 illustrates the positive correlation between financial sector development and Indonesia's economic growth from 1980 to 2018.

There are mixed findings from previous studies on the impacts of financial sector development on Indonesia's economic growth. For example, Majid and Mahrizal (2007) find that financial sector development (proxied by the ratio of total bank deposit liabilities to GDP) has no impact on Indonesia's economic growth from 1998 to 2016. Sumarni (2019) finds a positive but weak impact of financial sector development (proxied by the size of financial assets of the commercial bank) on Indonesia's economic growth from 2005 to 2016. They find that other proxies of financial sector development (i.e., the size of banking credit and third-party bank deposits) show insignificant results. Fukuda (2012) and Habibullah, 1998a, Habibullah, 1998b find that financial sector development (proxied by money supply to GDP ratio) has a positive impact on Indonesia's economic growth. The main concern here is that the authors use different proxies for financial sector development; thus, different proxies of financial sector development produce different results.

There is one issue often discussed in development economics: the presence of the “underground” or “shadow economy.” The shadow economy causes a country to underreport its true value of GDP. It also causes the government tax collection below what the number that government should be able to collect. Smith (1994) defines the “underground economy” as “market-based production of goods and services, whether legal or illegal, that escapes detection in the official estimates of GDP.” The “shadow economy” is often related to criminal activities (Naylor, 1996; Habibullah and Eng, 2006b). Schneider and Enste's (2002) definition of the “shadow economy” comprises “all economic activities that would generally be taxable if reported to the tax authorities.” The shadow economy reduces the overall tax revenue and hampers the government's capacity to provide public infrastructure and improve the welfare of the society. Therefore, mitigating the shadow economy is an important agenda for many governments (Din et al., 2019).

Kelmanson et al. (2021) suggest more comprehensive measures in combating shadow economy in emerging market economies in Europe. These measures include reducing regulatory and administrative burdens, promoting good governance, improve tax compliance, automating procedures and promoting electronic payments. The adoption of efficient electronic payment system through better equip financial institutions will be able to mitigate shadow economy. A study by Sangirova et al. (2021) found evidence that as electronic payments system increases, the level of shadow economy to GDP decreases proportionally in Uzbekistan. In another study, by using a sample of 29 developed and developing countries over the period of 1975–2015, Gharleghi and Jahanshahi (2020) found support to the contention that financial development has the ability to reduce the size of shadow economy.

In other studies, Ginevicius et al. (2020) exert that the national economic development can play a major role in reducing shadow economy. They conclude that “the higher the level of national economic development, the lower the size of the shadow economy.” On one hand, Esaku (2021) found that narrowing income inequality in Uganda can mitigate the size of shadow economy. Other factors that affect shadow economy in Uganda include inflation, government expenditure, openness, corruption, and democracy. On the other hand, Berdiev et al. (2020) postulate that health crisis has potential in affecting shadow economy. Using panel data on 130 countries, their study indicate that both the incidence and intensity of epidemics positively and significantly contribute to the spread of the shadow economy.

The shadow economy does exist in Indonesia. The Asian Development Bank (ADB) reports that the shadow economy contributed to the gross value added of the Yogyakarta and Banten provinces in 2011 by 37% and 27%, respectively. One of the reasons behind firms’ participation in the shadow economy is the difficulty to access the formal credit markets, particularly due to the complicated procedures for getting loans (ADB, 2011).

Rothenberg et al. (2016) find that 93% of firms in Indonesia participated in the shadow economy. They conclude that: (i) most firms participating in Indonesia's shadow economy were the micro, small, and medium enterprises (MSMEs) but with significant contribution to the economy; (ii) these firms paid low wages and exhibited low productivity; (iii) entrepreneurs in these firms had low educational attainment; and (iv) these firms are limited to local markets and rarely expand their business. Rothenberg et al. suggest that some of these firms will eventually leave the shadow economy if they have access to formal financial resources.

Recent studies by Saraswati and Agustina (2020) and Rezky (2020) indicate that the size of shadow economy is still high. Saraswati and Agustina (2020) stated that the size of shadow economy in Indonesia during the period 1981 to 2014 averaging 29% of gross domestic product. Their study found that human capital measures by life expectancy mitigate shadow economy; while enrolment in tertiary education induces expansion in shadow economy in Indonesia. On the other hand, Rezky (2020) found that for the period 2000 to 2017, the size of shadow economy is between 18% to 21% in Indonesia. Their findings suggest that tax burden, tight regulation, unemployment rate and corruptions affect the size of shadow economy.

Based on the findings from the studies by the ADB (2011) and Rothenberg et al. (2016), we induce a hypothesis that using financial sector development to provide greater access for the population to financial resources can reduce Indonesia's shadow economy. In this study, we investigate whether financial sector development can reduce the size of Indonesia's shadow economy. We calculate the size of Indonesia's shadow economy using the “Modified Cash to Deposits Ratio” (MCDR) method, which will be explained later in this paper.

We find a non-linear long-run relationship between financial sector development and the size of Indonesia's shadow economy. The size of Indonesia's shadow economy expands at the early stages of financial sector development to a turning point and decreases when financial sector development increases further.

Our paper proceeds as follows. Section 2 elaborates existing literature on the shadow economy. Section 3 explores previous studies that sought to measure the size of Indonesia's shadow economy. We also discuss our estimation of the size of Indonesia's shadow economy using the MCDR approach. Section 4 discusses the model in our study, the estimation methods, variables, and data sources. Section 5 analyzes the regression results. Section 6 concludes the paper.

## 2 Related literature

We can generally define the shadow economy as comprising activities that involved unreported or underreported income from the legal production of goods and services (Schneider and Enste, 2000). Previous studies find that various factors have caused people and firms to participate in the shadow economy. Among these factors are unemployment (Dell’Anno and Solomon, 2008), rules, regulations, taxes, and welfare benefits (Bajada and Schneider, 2005), weak governance (Friedman et al., 2000), crime rate (Wang et al., 2006), inflation (Bittencourt et al., 2014), and the level of financial sector development (Bose et al., 2012).

Our study examines factors that determine the size of Indonesia's shadow economy. We include unemployment, inflation, and the level of financial sector development as independent variables in our study. These macroeconomic variables are selected because we can use them to evaluate the impact of Indonesia's macroeconomic policies on the shadow economy. Besides these variables, we also include the following as independent variables: foreign direct investment (FDI), broad money supply (M2), and domestic banking credit to the private sector. Among these independent variables, the financial sector development variable is the main interest of our study.

Does financial sector development reduce the shadow economy? Findings from previous studies tend to support the premise that the financial sector development narrows the shadow economy. For example, Bayar and Ozturk (2016) find that both financial sector development and institutional quality reduced the shadow economy in the nine transition economies of the European Union in the 2003–2014 period. Berdiev and Saunoris (2016) find that financial sector development reduces the shadow economy in 161 countries over the 1960–2009 period. Henri (2018) finds that financial sector development curbed the shadow economy in 41 sub-Saharan African countries during 1991 and 2015. Bayar and Aytemiz (2017) find that financial sector development reduced Turkey's shadow economy during 1960 and 2009. Habibullah et al. (2016), Din (2016), and Din et al. (2019) and Habibullah et al. (2017) find that financial sector development had shrunken the size of Malaysia's shadow economy.

Some studies seek to explain how financial sector development helps to curb the shadow economy. For instance, Bose et al. (2012) find that firms in countries with a well-developed financial sector have easy access to external financing, particularly in the banking credit market. To apply for a loan from a bank, it is a common practice for a borrowing firm to declare its assets, a portion of which will serve as collateral for the loan. By such a declaration, the respective firm's assets will subject to government taxation.

Gordon and Li (2009) find that the value provided by the formal financial institution in countries that have a well-developed financial sector is considerably higher than the incentive to evade tax. Under such a circumstance, there is not much incentive for firms to enter the shadow economy. There is a higher incentive to evade tax in countries that have a less-developed financial sector. In these countries, access to the credit market is limited amid asymmetric information, limited loan supply, and high borrowing costs. Consequently, the collateral requirements for loans are higher than those in countries with a well-developed financial sector. Thus, a borrowing company in a country with a less-developed financial sector is subjecting itself to the risk of receiving higher taxation when it declares its assets. In this kind of environment, the size of the shadow economy can be substantial (Bose et al., 2012).

## 3 Estimating the size of Indonesia's shadow economy

Some studies attempted to estimate the size of Indonesia's shadow economy (see Table 1). For instance, Tan et al. (2017) estimated that the average size of Indonesia's shadow economy during 1984 and 2012 was at around 28% of the official GDP. This figure is higher than the 19.8% average estimate by Medina and Schneider (2018).

**Table 1:** Various estimates of the size of Indonesia's shadow economy 1980–2020.

Year	Elgin and Oztunali (2012)	Alm and Embaye (2013)	Tan et al. (2017)	Medina and Schneider (2018)	Wibowo& Sharma (2005)	Panjaitan (2007)	Nizar &Purnomo (2011)	Samuda (2016)	Azwar and Mulyawan (2017)	Ramadhan (2019)	Our study
1970	33.72										
1971	33.25										
1972	32.61										
1973	31.8					68.06					
1974	30.97					56.73					
1975	29.96					59.82					
1976	29.02				9.57	63.33					
1977	28.18				8.94	65.68					
1978	27.3				8.65	65.99					
1979	26.41				9.21	75.42					
1980	25.66				9.36	80.08					0
1981	24.82				8.52	79.07					6.4
1982	24.04				9.08	79.30					12.0
1983	23.31				9.73	56.85					18.2
1984	22.71	57.2	18.9		10.94	85.61					21.9
1985	22.17	55.2	21.3		16.17	87.36					29.0
1986	21.94	51.9	20.1		36.72	80.19					37.2
1987	21.48	52.6	24.7		25.93	85.38					39.8
1988	21.12	51.8	23.9		16.47	90.61					39.5
1989	20.79	52.1	25.4		15.79	96.54					35.4
1990	20.47	54.9	34.4		21.21	104.98					41.0
1991	20.12	50.4	32.4	22.69	22.30	98.49					37.5
1992	19.76	35.3	34.4	21.88	27.34	100.41					47.8
1993	19.44	29.3	34	22.08	31.80	87.78					50.0
1994	19.13	30.5	33.3	21.29	29.90	89.64					57.0
1995	18.79	28.2	34.5	20.32	35.38	88.77					54.8
1996	18.42	27.3	26.4	19.34	43.94	92.32					46.5
1997	18.05	30.4	27.4	19.19	40.54	99.89					50.4
1998	17.66	37.4	35	17.48	39.25	103.20					63.3
1999	17.81	35.1	16.7	20.03	46.41	115.94					82.1
2000	17.9	31.6	38.8	19.4		96.39	5.0			5.73	65.2
2001	17.96	32.5	36	19.75		107.88	6.0	9.64		6.41	71.6
2002	17.98	34.1	28.1	21.13		112.08	5.0	8.74		7.19	69.2
2003	18.05	34.3	31.3	21.6		99.27	6.0	7.94		7.79	70.3
2004	18.07	31.5	33.7	20.88		97.29	6.0	7.13		7.77	76.8
2005	18.05	35.1	33	20.52			6.0	8.28		7.61	81.6
2006	17.97	36.3	24.7	20.57			5.0	8.40		7.39	74.8
2007	17.9		23.7	19.83			6.0	8.17		7.49	67.1
2008	17.82		29.1	19.1			6.0	8.83		7.84	83.3
2009			23.3	19.99			5.0	5.86		8.00	76.7
2010			23.1	19.14				6.39		7.96	74.3
2011			24.8	18.35				7.01	20.65	7.83	73.2
2012			22.9	17.92				7.58	21.42	7.89	74.6
2013				17.62				9.89	22.34	7.72	81.2
2014				16.75					22.70	7.65	79.4
2015				17.46					23.39	7.74	79.4
2016										8.12	69.1
2017										8.36	72.5
2018											74.7
2019											71.5
2020											69.0

Sources: Elgin and Oztunali (2012), Alm and Embaye (2013), Tan et al. (2017), Medina and Schneider (2018), Wibowo and Sharma (2005), Panjaitan (2007), Nizar and Purnomo (2011), Samuda (2016) by calculating from the quarterly figures, Azwar and Mulyawan (2017) by averaging from the quarterly figures, and Ramadhan (2019). Elgin and Oztunali (2012) also provide the size of Indonesia'a shadow economy for the period 1960–1969 as 32.78, 33.08, 33.08, 33.13, 33.37, 33.59, 33.66, 33.71, 33.9 and 33.94, respectively.

**Figure 2:**
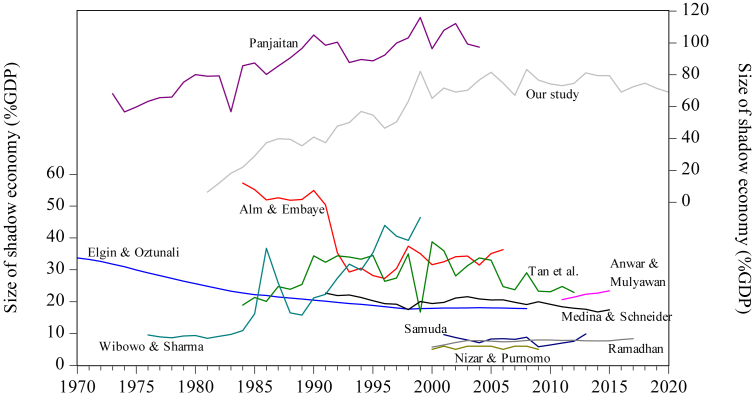
Trends of the size of the shadow economy by various authors, 1980–2020.

Figure 2 displays the estimates and trends of the size of Indonesia's shadow economy by various authors. For example, Elgin and Oztunali (2012) suggested that the size of Indonesia's shadow economy shrunk from 34% of the official GDP in 1970 to 18% in 2008. Alm and Embaye (2013) estimated that Indonesia's shadow economy narrowed from 57% of the official GDP in 1984 to 36% in 2006. Wibowo and Sharma (2005) suggested that the size of Indonesia's shadow economy expanded from 9.6% of the official GDP in 1976 to 46.4% in 1999. Panjaitan (2007) suggested that the size of Indonesia's shadow economy rose from 68% of the official GDP in 1973 to 97% in 2004.

We apply a different approach to estimate the size of Indonesia's shadow economy. We use the modified cash-to-deposit ratio (MCDR) method developed by Pickhardt and Sarda (2011, 2015) to estimate the size of the shadow economy. Habibullah et al. (2016), Din (2016), Habibullah et al. (2017), and Din et al. (2019) use the MCDR method for studies on Malaysia's shadow economy. The MCDR method sets the size of the shadow economy to zero in the base year. It assumes that excess cash holdings in the base year are fully attributed to the shadow economy. The MCDR method measures all cash used in illicit economic activities.

Rasbin (2013) reports that the illegal activities involved in the underground Indonesian economy in the year 2002 include illegal exports of sand dredged from the sea, wood products, and sea wildlife; import of illegal electronics products; prostitution; and gambling. Therefore, the MCDR method produces a higher estimate of the size of the shadow economy than the estimate from the currency demand method.

Based on the MCDR approach, the ratio of income generated by the shadow economy to the official income for Indonesia is formulated as follows:

(1) $$\frac{{\text{CC}}_{\text{t}}-{\text{CC}}_{0}}{{\text{CC}}_{0}+{\text{DD}}_{\text{t}}}=\frac{{\text{Y}}_{\text{Ut}}}{{\text{Y}}_{\text{Lt}}}$$

where ${\text{CC}}_{\text{t}}$ is the currency in circulation at the end of the year t; ${\text{CC}}_{0}$ is the currency in circulation at the end of the base year, which is 1980 here; ${\text{DD}}_{\text{t}}$ is the demand deposits at the end of the year t; ${\text{Y}}_{\text{Lt}}$ and ${\text{Y}}_{\text{Ut}}$ denote the size of the legal and shadow economy, respectively. Therefore, ${\text{Y}}_{\text{Ut}}/{\text{Y}}_{\text{Lt}}$ measures the share of the shadow economy to the legal economy (official GDP).

Table 1 shows our estimates for the size of Indonesia's shadow economy for the 1980–2020 periods. Figure 2 displays our estimates in a graph. We estimate that the size of Indonesia's shadow economy rose from 6.4% in 1981 to 69.0% in 2020, averaging 58.1% for 40 years.

Having estimated the size of the shadow economy for Indonesia for the 1980–2020 periods, we investigate factors affecting Indonesia's shadow economy. Specifically, we examine whether empirical evidence supports the claim that financial sector development reduces the shadow economy. Other factors included in our model serve as control variables.

## 4 Modeling Indonesia's shadow economy

### 4.1 Model specification

Our model for Indonesia's shadow economy is specified as the following:

(2) $${\text{se}}_{\text{t}}={\text{θ}}_{0}+{\text{θ}}_{1}{\text{rgdppc}}_{\text{t}}+{\text{θ}}_{2}{\text{findev}}_{\text{t}}+{\text{θ}}_{3}{\text{findev}}_{\text{t}}^{2}+{\text{θ}}_{4}{\text{fdi}}_{\text{t}}+{\text{θ}}_{5}{\text{misery}}_{\text{t}}+{\text{ε}}_{\text{t}}$$

Where ${\text{se}}_{\text{t}}$ is the size of the shadow economy (stated as a percentage to GDP); ${\text{rgdppc}}_{\text{t}}$ is the real GDP per capita; ${\text{misery}}_{\text{t}}$ is the misery index, ${\text{fdi}}_{\text{t}}$ is the net foreign direct investment to GDP ratio; ${\text{findev}}_{\text{t}}$ is the financial sector development indicator; and ${\text{findev}}_{\text{t}}^{2}$ is the financial sector development indicator stated in a squared form to incorporate the possibility of a non-linear relationship between financial sector development and the shadow economy. All variables are stated in natural logarithm forms.

If the relationship between financial sector development and the shadow economy exhibits an inverted U-shape curve, we would expect ${\text{θ}}_{2}>0$ and ${\text{θ}}_{3}<0$. It implies that the shadow economy expands in the early stage of financial sector development to a turning point, after which a higher level of financial sector development shrinks the shadow economy. For other parameters, we expect ${\text{θ}}_{1}>0,{\text{θ}}_{5}>0,$ and ${\text{θ}}_{4}<0$. The error term ${\text{ε}}_{\text{t}}$ is expected to have a zero mean and a constant variance. We use two proxies for the financial sector development indicator: (1) the ratio of money supply M2 to GDP; (2) the ratio of domestic credit to the private sector to GDP.

The real GDP per capita has a rather ambiguous impact on the shadow economy. Pickhardt and Sarda (2015) point out that the sign of the real GDP per capita parameter depends on the structure of the shadow economy. Some studies on the developed economies find a negative relation between the real GDP per capita and the shadow economy (Schneider, 2005). Bajada (2003), Giles (1997), Ferreira-Tiryaki (2008), and Granda-Carvajal (2010) find a positive relationship between the shadow economy and output. In this study, we expect the real GDP per capita to increase the size of Indonesia's shadow economy. We assume that rapid economic growth widens the income disparity between the rich and the poor. The poor are compelled to work in the shadow economy to support the rising living standards and prices stemming from economic growth.

Some studies find that FDI stimulates economic growth through spillover effects, such as technology transfers, capital accumulation, improvement in per capita income, improvement in productivity, export promotion, and human capital development (Opoku et al., 2019; Almasaied et al., 2008). FDI is also an important source of tax revenue for the government in many countries. FDI tends to flow to countries with better quality of institutions, better governance, and stronger protection of property rights (Lee et al., 2018; Hyunh et al., 2019). FDI creates employment opportunities and improves the quality of institutions, which helps to reduce the shadow economy. Nikopour et al. (2009), Davidescu (2015), and Hyunh et al. (2019) find that FDI curbs the shadow economy.

The misery index measures the “hardship” suffered by a country's population. It is calculated as the summation of the inflation rate and the unemployment rate. Inflation weakens households' purchasing power, while unemployment causes households to lose their income. Higher inflation and unemployment rate expand the shadow economy because more people participate in underground activities to support their livelihood. Studies by Dell’Anno and Solomon (2008), Sahnoun and Abdennadher (2019), and Bajada (2003) find a positive relationship between unemployment and the shadow economy. Meanwhile, studies by Mazhar and Meon (2017) and Baklouti and Boujelbene (2019) show that inflation induces the shadow economy. Bittencourt et al. (2014) conclude that increase financial sector development and lower inflation curbs size of the shadow economy.

### 4.2 Estimation method

We apply various estimation methods to our model: (1) the Ordinary Least Square (OLS); (2) the Dynamic OLS (DOLS); (3) the Fully Modified OLS (FMOLS); (4) the Canonical Cointegrating Regression (CCR); and (5) the Autoregressive distributed lag (ARDL). The OLS is the simplest among these methods but can produce a robust standard error (Newey and West, 1987). The DOLS, the FMOLS, and the CCR are more efficient and robust than the OLS, particularly for small samples and to work with models with heteroscedasticity, autocorrelation, and non-normality of errors. The ARDL is a robust method to estimate models with different order of stationarity on its variables, small-sample properties, and endogeneity problem. Habibullah et al. (2016) elaborates on the advantages and disadvantages of these five estimation methods.

All variables in a time series model should be stationary (i.e., they do not have unit roots) to produce non-spurious estimation results. Using the Eliott et al. (1996) method to perform the unit root tests, we find that most variables in our model are not stationary at levels (i.e., not I (0)). Hence, we cannot estimate our model before resolving the non-stationarity problem. The first step to dealing with a model that involves non-stationary variables is to ensure that the model is cointegrated. A model is cointegrated if there is a long-term relationship among variables in the respective model. If the model is cointegrated, we can safely estimate our model despite the presence of non-stationary variables. If the model is not cointegrated, the estimation will produce spurious results and invalidate the hypothesis testing.

We must apply the appropriate cointegration test before using our chosen estimation method. We use the two-step Engle-Granger cointegration test for the OLS estimation, the Hansen instability test for the FMOLS, DOLS, and CCR; and the Bound F-test for the ARDL.

All variables in our models must be non-stationary at the same order of integration before we can implement the EG cointegration procedure. We use the Eliott et al. (1996) method to perform the unit root test. In the first step of the EG procedure, we estimate our model to obtain the estimation residuals. In the second step, the residuals are regressed on the lagged residuals (Faik, 1998). If the residuals are stationary, we can conclude that the model is cointegrated.

The Hansen instability test starts with the premise that a cointegrated model should have stable parameters. Therefore, a non-cointegrated model shows evidence of parameter instability. The null hypothesis in the Hansen test is the existence of cointegration, while the alternative hypothesis is no cointegration. Hansen (1992) proposes the Lc statistic test to evaluate the stability of the parameters (EViews 12, 2020). For further explanation on the Hansen instability test, see Hansen (1992).

In the case of ARDL, we use the Bound F-test statistics to examine the existence of cointegration by estimating the following conditional error-correction model (ECM):

$$\Delta {\text{se}}_{\text{t}}={\text{α}}_{0}+{\text{α}}_{1}{\text{se}}_{\text{t}-1}+{\text{α}}_{2}{\text{rgdppc}}_{\text{t}-1}+{\text{α}}_{3}{\text{findev}}_{\text{t}-1}+{\text{α}}_{4}{\text{findev}}_{\text{t}-1}^{2}+{\text{α}}_{5}{\text{fdi}}_{\text{t}-1}$$

$$+{\text{α}}_{6}{\text{misery}}_{\text{t}-1}+\overset{\text{p}}{\underset{\text{i}=1}{\sum }}{\text{γ}}_{1\text{i}}\Delta {\text{se}}_{\text{t}-\text{i}}+\overset{\text{q}}{\underset{\text{i}=0}{\sum }}{\text{γ}}_{2\text{i}}\Delta {\text{rgdppc}}_{\text{t}-\text{i}}+\overset{\text{r}}{\underset{\text{i}=0}{\sum }}{\text{γ}}_{3\text{i}}\Delta {\text{findev}}_{\text{t}-\text{i}}$$

(3) $$+\overset{\text{s}}{\underset{\text{i}=0}{\sum }}{\text{γ}}_{4\text{i}}\Delta {\text{findev}}_{\text{t}-1}^{2}+\overset{\text{v}}{\underset{\text{i}=0}{\sum }}{\text{γ}}_{5\text{i}}\Delta {\text{fdi}}_{\text{t}-\text{i}}+\overset{\text{w}}{\underset{\text{i}=0}{\sum }}{\text{γ}}_{6\text{i}}\Delta {\text{misery}}_{\text{t}-\text{i}}+\varepsilon_{\text{t}}$$

The null hypothesis of no cointegration is stated as ${\text{α}}_{1}={\text{α}}_{2}={\text{α}}_{3}={\text{α}}_{4}={\text{α}}_{5}={\text{α}}_{6}=0$, whereas the alternative hypothesis is stated as ${\text{α}}_{1}\neq {\text{α}}_{2}\neq {\text{α}}_{3}\neq {\text{α}}_{4}\neq {\text{α}}_{5}\neq {\text{α}}_{6}\neq 0$. We reject the null hypothesis if the computed F-statistic exceeds the upper bounds of the critical value. Rejection of the null hypothesis shows that the model is cointegrated. This implies that the long-run model in Eq. (2) valid. Eq. (3) must pass the non-serial correlation test with optimum lag length chosen using the Schwartz criteria suggested by Pesaran et al. (2001).

We complement our OLS regression with the quantile regression to produce a more comprehensive analysis. The quantile regression allows the regressors to affect the conditional distribution of the dependent variable (Koenker and Basset, 1978). To be more specific, the quantile regression permits the estimated parameters (slopes) to have different values at different points of the dependent variable's conditional distribution. Because the quantile regression is a nonparametric regression, it does not impose any functional form on the shadow economy relationship. Furthermore, the quantile regression is not sensitive to the outliers.

The quantile regression is formulated as follows:

(4) $${\text{se}}_{\text{t}}={\text{x}}_{\text{t}}^{\text{′}}{\text{β}}_{\text{τ}}+{\text{μ}}_{\text{τt}}\;0<\text{τ}<1$$

(5) $${\text{Quantile}}_{\text{τ}}\left({\text{se}}_{\text{t}}|{\text{x}}_{\text{t}}\right)={\text{x}}_{\text{t}}^{\text{′}}{\text{β}}_{\text{τ}}$$

Where ${\text{x}}_{\text{t}}^{\text{′}}$ is the vector of explanatory variables and ${\text{β}}_{\text{τ}}$ is the vector of parameters associated with the τ-th percentile, while ${\text{μ}}_{\text{τt}}$ is the unknown error term.

The ${\text{Quantile}}_{\text{τ}}\left({\text{se}}_{\text{t}}|{\text{x}}_{\text{t}}\right)={\text{x}}_{\text{t}}^{\text{′}}{\text{β}}_{\text{τ}}$ equals the τ-th conditional quantile of se given x with τ∈(0,1). The quantile regression allows different parameters across different quantiles to have different values of τ.

The τ-th quantile regression estimates ${\text{β}}_{\text{τ}}$ by solving the following minimization problem.

(6) $$\hat{\text{β}}\left(\text{τ}\right)={\text{arg ​min}}_{\text{β}}\left[\underset{\left\{{\text{se}}_{\text{t}}\geq {\text{x}}_{\text{t}}^{\text{′}}\text{β}\right\}}{\sum }\text{τ}\left|{\text{se}}_{\text{t}}-{\text{x}}_{\text{t}}^{\text{′}}\text{β}\right|+\underset{\left\{{\text{se}}_{\text{t}}<{\text{x}}_{\text{t}}^{\text{′}}\text{β}\right\}}{\sum }\left(1-\text{τ}\right)|{\text{se}}_{\text{t}}-{\text{x}}_{\text{t}}^{\text{′}}\text{β}|\right].$$

The median of the regression is achieved when τ=0.5 and the coefficients of the absolute values are all equal to one.

### 4.3 Data sources

Our study uses annual data that spans from 1980 to 2020. Currency in circulation and demand deposits data are obtained from the Asian Development Bank's Key Indicators for Asia and the Pacific publications from various years (https://www.adb.org/publications/series/key-indicators-for-asia-and-the-pacific). Data on the gross domestic product (GDP), real GDP per capita (2010 = 100), foreign direct investment (FDI) net inflow, broad money supply (M2), domestic credit to the private sector, inflation, and unemployment rates (for the misery index) are compiled from the World Bank's database (World Development Indicator, http://data.worldbank.org/indicator). The FDI net inflows, M2, and domestic credit to private sector variables are expressed as ratios to GDP. The misery index is defined as the summation of inflation and unemployment rate. The misery index is used to measure the “hardship” experienced by a country's population. All variables are transformed into logarithm by using Busse and Hefeker (2007) approach.

## 5 Results and analysis

### 5.1 The OLS, FMLS, DOLS, ARDL, and CCR regressions

The unit root test results for the DF-GLS procedure indicate that all variables are stationary after the first-order differencing, that is I (0) (see Table 2).

**Table 2:** DF-GLS unit root test results.

Variable	Level:		First-difference:	
	Intercept	Intercept + trend	Intercept	Intercept + trend
${\text{se}}_{\text{t}}$	−0.8934 (0)	−1.8646 (0)	−2.3207∗∗(0)	−4.3535∗∗(0)
${\text{rgdppc}}_{\text{t}}$	0.5732 (1)	−2.6094 (1)	−4.7186∗∗(0)	−4.6018∗∗(0)
${\text{domcredit}}_{\text{t}}$	−1.2257 (0)	−1.9027 (1)	−4.0705∗∗(0)	−4.4631∗∗(0)
${\text{domcredit}}_{\text{t}}^{2}$	−1.3348 (1)	−1.9464 (1)	−4.3296∗∗(0)	−4.5852∗∗(0)
$\text{m}2_{\text{t}}$	−0.5926 (1)	−1.4852 (1)	−3.1925∗∗(0)	−3.2744∗∗(0)
$\text{m}2_{\text{t}}^{2}$	−0.6439 (1)	−1.5167 (1)	−3.2264∗∗(0)	−3.2696∗∗(0)
${\text{fdi}}_{\text{t}}$	−2.2824 (0)	−2.4121 (0)	−4.8616∗∗(0)	−5.2875∗∗(0)
${\text{misery}}_{\text{t}}$	−1.7998 (1)	−2.6555 (1)	−6.0276∗∗(0)	−6.8092∗∗(0)

Notes: ∗∗ indicates that the variable is statistically significant at 5% significance level. The figures in round (…) brackets are Schwarz information criterion automatic lag length truncation.

Table 3 shows that cointegration is detected in the case of OLS, ARDL, DOLS, and CCR estimates. The null hypothesis of non-cointegration is rejected for the OLS and ARDL, while the null hypothesis of cointegration cannot be rejected for the DOLS and CCR. The E-G test statistic, Bound F-test statistic, and ${\text{ect}}_{\text{t}-1}$ t-statistic are significant at the 1% level, while the ${\text{L}}_{\text{c}}$ statistic is insignificant for DOLS and CCR. Hence, the long-run estimates from the OLS, ARDL, DOLS, and CCR methods on our model are non-spurious.

**Table 3:** Estimation Results of the Long-run Model for the Shadow Economy With the M2/GDP Ratio as a Proxy for Financial Sector Development (t-statistics values are in the round brackets) [p-values are in the square brackets].

Estimators	Intercept	${\text{rgdppc}}_{\text{t}}$	${\text{m}2}_{\text{t}}$	${\text{m}2}_{\text{t}}^{2}$	${\text{fdi}}_{\text{t}}$	${\text{misery}}_{\text{t}}$
OLS (robust estimates)	−39.562∗∗∗ (−4.5990)	0.6211∗∗∗ (2.8959)	17.720∗∗∗ (3.9837)	−1.9857∗∗∗ (−3.8521)	−0.1114∗∗ (−2.6665)	−0.0581 (−0.8373)
	E-G test: -4.275∗∗∗		SER = 0.197	${\bar{\text{R}}}^{2}$ = 0.875	Optimal point = 4.46	
ARDL (1,4,1,4,0,1)	−32.052∗∗∗ (−9.0633)	0.3064∗∗∗ (5.8079)	15.822∗∗∗ (9.4985)	−1.7867∗∗∗ (−9.2475)	0.1340∗∗ (3.6562)	−0.1041∗∗∗ (−4.9515)
	Bounds F-test: 10.11∗∗∗		SER = 0.072	${\bar{\text{R}}}^{2}$ = 0.974	Optimal point = 4.43	
	${\text{ect}}_{\text{t}-1}$ = -0.8393∗∗∗					
FMOLS {Prewhitening lag = 1}	−43.748∗∗∗ (−7.6514)	0.6567∗∗∗ (4.3174)	21.892∗∗∗ (6.7186)	−2.4621∗∗∗ (−6.3876)	−0.1255∗∗∗ (−2.7641)	−0.1200∗ (−1.8699)
	${\text{L}}_{\text{c}}$ = [1.407]∗∗∗		SER = 0.217	${\bar{\text{R}}}^{2}$ = 0.849	Optimal point = 4.45	
DOLS {lead = 1, lag = 1}	−42.260∗∗∗ (−8.7259)	0.4631∗∗∗ (3.7773)	19.572∗∗∗ (7.5817)	−2.1816∗∗∗ (−6.9338)	0.1409∗∗ (2.1309)	−0.1155∗∗ (−2.1376)
	${\text{L}}_{\text{c}}$ = [0.071]		SER = 0.101	${\bar{\text{R}}}^{2}$ = 0.967	Optimal point = 4.49	
CCR {Prewhitening lag = 1}	−35.920∗∗∗ (−6.8655)	0.6734∗∗∗ (4.0863)	15.618∗∗∗ (5.5236)	−1.7149∗∗∗ (−5.0545)	−0.0620 (−1.0892)	−0.1011 (−1.3946)
	${\text{L}}_{\text{c}}$ = [0.653]		SER = 0.213	${\bar{\text{R}}}^{2}$ = 0.854	Optimal point = 4.55	

Notes: ∗∗∗, ∗∗ and ∗ indicate that the variable is statistically significant at 1%, 5% and 10% significance level, respectively. SER is the standard error of regression. The E-G test denotes the DF t-statistic of the cointegrating regression's residuals. ${\text{L}}_{\text{c}}$− statistic measures Hansen parameter instability test for cointegration. The E-G tests the null hypothesis of no cointegration. The Hansen test the null hypothesis of cointegration. The optimal point is calculated as $-{\hat{\text{θ}}}_{2}/2{\hat{\text{θ}}}_{3}$.

Results in Table 3 show that all variables in the long-run models are significant at the 5% significance level (*α*), except for the FDI and the misery index in the CCR estimation. The signs of all coefficients meet our expectations. The estimated coefficients for the financial sector development indicator (i.e., M2 to GDP ratio) show that ${\hat{\text{θ}}}_{2}>0$ and ${\hat{\text{θ}}}_{3}<0$, thus portraying an inverted U-shape curve. These findings are similar to the findings from Bose et al. (2012) that the shadow economy expands at a lower level of financial sector development until a certain point before declining afterward. The saturation points beyond which the shadow economy starts to decline is 4.46 for the OLS, 4.31 for the ARDL, 4.49 for the DOLS, and 4.55 for the CCR. Thus, our results show a non-linear long-term relationship between financial sector development and Indonesia's shadow economy.

When using the real GDP per capita to proxy the income variable, we find that the income variable has a positive impact on Indonesia's shadow economy. It implies that higher economic growth does not curtail Indonesia's shadow economy but rather causes people or firms to engage in the shadow economy. Because economic growth does not necessarily create a more equal income distribution, people and firms are still lured to enter the shadow economy whenever unemployment and inflation increase. It is a common phenomenon in developing economies, including in Indonesia where the poverty rate is more than 9% and the income disparity is widening. Nonetheless, our results using misery index does not support this contention. The impact of FDI on the size of Indonesia's shadow economy is negative. An increase in FDI inflows to Indonesia reduces the shadow economy.

Table 4 shows the cointegration test results of the model. In this case, the ratio of domestic credit to the private sector to GDP serves as a proxy for the level of financial sector development. The results show that cointegration is found in the case of the OLS, ARDL, and DOLS. The signs of all coefficients are in line with our expectations, except for the negative sign for the misery index in the OLS, ARDL and DOLS. All variables are significant in the case of ARDL estimation. All variables are significant in the OLS estimation, except for the misery index. Except for FDI, all other variables are significant in the DOLS estimation. The domcredit and the domcredit^2^ variables (both to proxy financial sector development) are also not significant with expected sign.

**Table 4:** Estimation Results of the Long-run Model for the Shadow Economy With the Domestic Credit/GDP Ratio as a Proxy for Financial Sector Development (t-statistics values are in the round brackets) [p-values are in the square brackets].

Estimators	Intercept	${\text{rgdppc}}_{\text{t}}$	${\text{domcredit}}_{\text{t}}$	${\text{domcredit}}_{\text{t}}^{2}$	${\text{fdi}}_{\text{t}}$	${\text{misery}}_{\text{t}}$
OLS (robust estimates)	−28.445∗∗∗ (−6.3222)	0.8352∗∗∗ (4.0275)	12.689∗∗∗ (4.6204)	−1.4918∗∗∗ (−4.4404)	−0.1946∗∗ (−2.6202)	−0.1193 (−1.5553)
	E-G test: -2.598∗∗		SER = 0.224	${\bar{\text{R}}}^{2}$ = 0.839	Optimal point = 4.25	
ARDL (1,5,5,5,5,3)	−23.806∗∗∗ (−8.9186)	0.8706∗∗∗ (6.8321)	10.683∗∗∗ (7.9507)	−1.2777∗∗∗ (−8.1184)	−0.3955∗∗∗ (−4.2587)	−0.2324∗∗∗ (−5.5454)
	Bounds F-test: 5.192∗∗∗		SER = 0.073	${\bar{\text{R}}}^{2}$ = 0.973	Optimal point = 4.18	
	${\text{ect}}_{\text{t}-1}$ = -1.431∗∗∗					
FMOLS {Prewhitening lag = 1}	−31.111∗∗∗ (−12.151)	0.7420∗∗∗ (5.8177)	14.418∗∗∗ (9.8505)	−1.7027∗∗∗ (−9.5837)	−0.2088∗∗∗ (−5.0536)	−0.1346∗∗ (−2.3901)
	${\text{L}}_{\text{c}}$ = [5.370]∗∗∗		SER = 0.229	${\bar{\text{R}}}^{2}$ = 0.832	Optimal point = 4.23	
DOLS {lead = 1, lag = 1}	−21.985∗∗∗ (−4.4924)	0.7203∗∗∗ (4.6947)	10.538∗∗∗ (4.7058)	−1.2787∗∗∗ (−4.9025)	−0.1497 (−1.5138)	−0.2068∗∗ (−2.8251)
	${\text{L}}_{\text{c}}$ = [0.053]		SER = 0.113	${\bar{\text{R}}}^{2}$ = 0.960	Optimal point = 4.12	
CCR {Prewhitening lag = 1}	−29.594∗∗∗ (−15.815)	0.7586∗∗∗ (5.7207)	13.608∗∗∗ (12.230)	−1.6056∗∗∗ (−11.892)	−0.1949∗∗∗ (−6.5130)	−0.1349∗ (−1.9695)
	${\text{L}}_{\text{c}}$ = [1.942]∗∗∗		SER = 0.227	${\bar{\text{R}}}^{2}$ = 0.835	Optimal point = 4.24	

Notes: ∗∗∗, ∗∗ and ∗ indicate that the variable is statistically significant at 1%, 5% and 10% significance level, respectively. SER is the standard error of regression. The E-G test denotes the DF t-statistic of the cointegrating regression's residuals. The ${\text{L}}_{\text{c}}$− statistic measures Hansen parameter instability test for cointegration. The E-G tests the null hypothesis of no cointegration. The Hansen test the null hypothesis of cointegration.

Results in Table 4 indicate that financial sector development has a non-linear relationship with Indonesia's shadow economy, where ${\hat{\text{θ}}}_{2}>0$ and ${\hat{\text{θ}}}_{3}<0$ in the OLS, the DOLS, and the ARDL cases. In the case of the domcredit variable, the turning point is 4.25 for the OLS, 4.18 for ARDL and 4.12 for the DOLS.

### 5.2 Quantile regression results

Table 5 shows the descriptive statistics of variables used in our quantile regression. The means of the variables are close to the medians. The skewness measure is negative for most of the variables, thus making the time series are skewed to the left. The kurtosis statistic is more than 3 for se (the size of shadow economy), m2 (M2 to GDP ratio), the FDI, and the misery index, demonstrating that these series have flatter tails compared to the normal distribution. The Jarque-Bera test suggests that the null hypothesis of normality is rejected for these variables, except for rgdppc, domcredit, and domcredit^2^.

**Table 5:** Descriptive Statistics of the Variables in the Model [p-values are in the round brackets].

Variables	Mean	Median	Std. dev	Skewness	Kurtosis	Jarque-Bera[p-values]	Anderson-Darling[p-values]
${\text{se}}_{\text{t}}$	4.65	4.91	0.56	−1.98	6.99	[0.000]∗∗∗	[0.000]∗∗∗
${\text{rgdppc}}_{\text{t}}$	8.32	8.29	0.38	0.07	1.94	[0.386]	[0.340]
$\text{m}2_{\text{t}}$	4.34	4.39	0.30	−1.07	3.56	[0.016]∗∗	[0.000]∗∗∗
$\text{m}2_{\text{t}}^{2}$	18.95	19.23	2.51	−0.92	3.32	[0.055]∗	[0.001]∗∗∗
${\text{domcredit}}_{\text{t}}$	4.12	4.09	0.41	−0.24	2.35	[0.577]	[0.290]
${\text{domcredit}}_{\text{t}}^{2}$	17.13	16.76	3.34	−0.05	2.15	[0.542]	[0.243]
${\text{fdi}}_{\text{t}}$	0.75	0.96	0.86	−1.09	3.87	[0.010]∗∗	[0.007]∗∗∗
${\text{misery}}_{\text{t}}$	3.53	3.26	0.66	1.34	3.61	[0.001]∗∗∗	[0.000]∗∗∗

Notes: ∗∗∗,∗∗, and ∗ indicate that the variable is statistically significant at 1%, 5% and 10% significance level, respectively.

**Figure 3:**
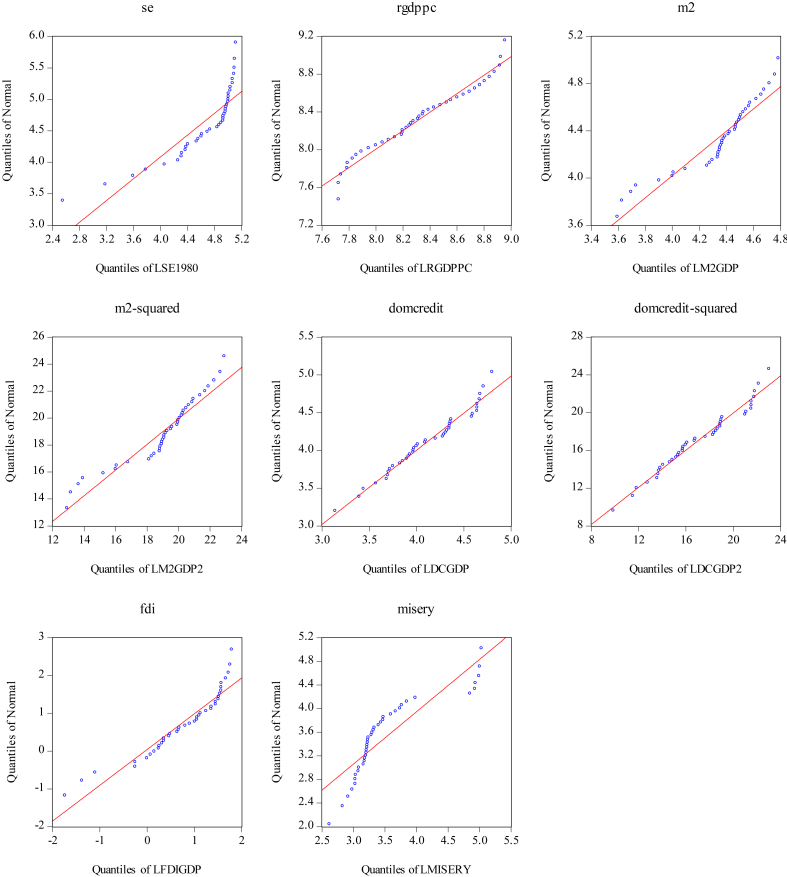
Quantile-quantile (Q–Q) plots for variables in the model.

Figure 3 shows the quantile-quantile (Q-Q) plots for the variables in our study. The Q-Q plots show that none of the variables present a good fit to normal distributions. As a matter of facts, Table 5 shows that the Anderson and Darling (1954) goodness of fit test rejected the null hypothesis of normality for almost all variables (the exceptions are the rgdppc and domcredit variables). Thus, most variables in our model do not follow the normal distribution.

Table 6 presents the quantile regression results for different proxies of the financial sector development indicator. Panel A for the M2 to GDP ratio, while Panel B for the ratio of domestic credit to the private sector to GDP. We run the quantile regressions for the 20^th^, 30^th^, … 70^th^, and 80^th^ quantiles. The pseudo ${\text{R}}^{2}$ is a measure of goodness of fit for each quantile. The pseudo ${\text{R}}^{2}$ decreases from the lower to the higher quantiles in both panels. It implies that the model can explain better about Indonesia's shadow economy in the lower quantiles than in the higher quantiles.

**Table 6:** Results of the Quantile Regressions (t-statistics values are in the round brackets) [p-values are in the square brackets].

Independent variables	Quantiles:						
	Q(0.20)	Q(0.30)	Q(0.40)	Q(0.50)	Q(0.60)	Q(0.70)	Q(0.80)
**Panel A: M2/GDP Ratio as a Proxy for Financial Sector Development**							
Constant	−51.777∗∗∗ (−5.1247)	−42.245∗∗∗ (−4.1762)	−42.645∗∗∗ (−3.6218)	−43.335∗∗∗ (−3.6650)	−33.101∗∗ (−2.4532)	−41.696∗∗∗ (−3.3975)	−32.776∗ (−1.8822)
${\text{rgdppc}}_{\text{t}}$	0.8188∗∗∗ (3.9711)	0.7479∗∗∗ (4.3698)	0.7281∗∗∗ (3.2033)	0.7048∗∗∗ (3.0372)	0.7212∗∗∗ (3.2382)	0.5483∗ (1.9759)	0.4590 (1.4075)
$\text{m}2_{\text{t}}$	22.781∗∗∗ (4.2489)	18.846∗∗∗ (3.7734)	19.017∗∗∗ (3.2196)	19.428∗∗∗ (3.2706)	14.638∗∗ (2.1558)	19.031∗∗∗ (3.1847)	15.100∗ (1.8230)
$\text{m}2_{\text{t}}^{2}$	−2.5737∗∗∗ (−3.999)	−2.1489∗∗∗ (−3.678)	−2.1549∗∗∗ (−3.111)	−2.2029∗∗∗ (−3.164)	−1.6509∗∗ (−2.080)	−2.1681∗∗∗ (−3.073)	−1.6829∗ (−1.702)
${\text{fdi}}_{\text{t}}$	−0.1165∗∗∗ (−2.5865)	−0.1179∗∗∗ (−2.6494)	−0.1351∗∗ (−2.1825)	−0.1325∗∗ (−2.1270)	−0.1258∗ (−1.9722)	−0.0881 (−1.4092)	−0.0415 (−0.3743)
${\text{misery}}_{\text{t}}$	−0.1798∗∗ (−2.5160)	−0.1253∗ (−1.8427)	−0.1203 (−1.3544)	−0.1173 (−1.3171)	−0.1174 (−1.5420)	0.1085 (0.4286)	0.0252 (0.1414)
${\text{Pseudo ​R}}^{2}$	0.720	0.706	0.690	0.655	0.602	0.557	0.514
SER	0.299	0.229	0.204	0.204	0.220	0.255	0.263
Wald asymmetric test				11.399 [0.876]			
Wald slope equality test				26.680 [0.640]			
Optimal point	4.43	4.39	4.41	4.41	4.43	4.39	4.49
**Panel B: Domestic Credit/GDP Ratio as a Proxy Financial Sector Development**							
Constant	−27.953∗∗∗ (−6.7867)	−27.989∗∗∗ (−7.5406)	−28.049∗∗∗ (−7.3114)	−28.930∗∗∗ (−7.2191)	−23.765∗ (−1.7786)	−27.085∗∗∗ (−3.6705)	−26.152∗∗ (−2.2256)
${\text{rgdppc}}_{\text{t}}$	0.7336 (1.5961)	0.9341∗∗∗ (5.0667)	0.8926∗∗∗ (4.3868)	0.9470∗∗∗ (4.2925)	0.9655∗∗∗ (3.9202)	0.9972∗∗∗ (4.3944)	1.0171∗∗∗ (4.0373)
${\text{domcredit}}_{\text{t}}$	12.800∗∗∗ (4.8497)	12.036∗∗∗ (5.5574)	12.183∗∗∗ (5.3850)	12.418∗∗∗ (5.1852)	9.754 (1.4141)	11.265∗∗∗ (2.9058)	10.444∗ (1.7622)
${\text{domcredit}}_{\text{t}}^{2}$	−1.5120∗∗∗ (−4.5242)	−1.4009∗∗∗ (−5.2172)	−1.4123∗∗∗ (−5.0879)	−1.4388∗∗∗ (−4.8947)	−1.1265 (−1.3892)	−1.3178∗∗∗ (−2.8607)	−1.2184∗ (−1.7581)
${\text{fdi}}_{\text{t}}$	−0.0542 (−0.1578)	−0.2265∗∗ (−2.3749)	−0.2194∗∗ (−2.2968)	−0.2844∗∗∗ (−3.0357)	−0.2651∗∗ (−2.6493)	−0.2261∗∗ (−2.4665)	−0.1592∗∗ (−2.2743)
${\text{misery}}_{\text{t}}$	−0.1300 (−0.7138)	−0.1831∗ (−2.0134)	−0.1746∗ (−1.8660)	−0.1758∗ (−1.8427)	−0.0745 (−0.6130)	−0.0362 (−0.1491)	0.1472 (0.4530)
${\text{Pseudo ​R}}^{2}$	0.715	0.679	0.639	0.571	0.498	0.423	0.366
SER	0.314	0.256	0.243	0.239	0.247	0.265	0.394
Wald asymmetric test				8.755 [0.965]			
Wald slope equality test				16.351 [0.979]			
Optimal point	4.23	4.30	4.31	4.32	4.33	4.27	4.29

Notes: ∗∗∗, ∗∗ and ∗ indicate that the variable is statistically significant at 1%, 5% and 10% significance level, respectively. SER is the standard error of regression.

**Figure 4:**
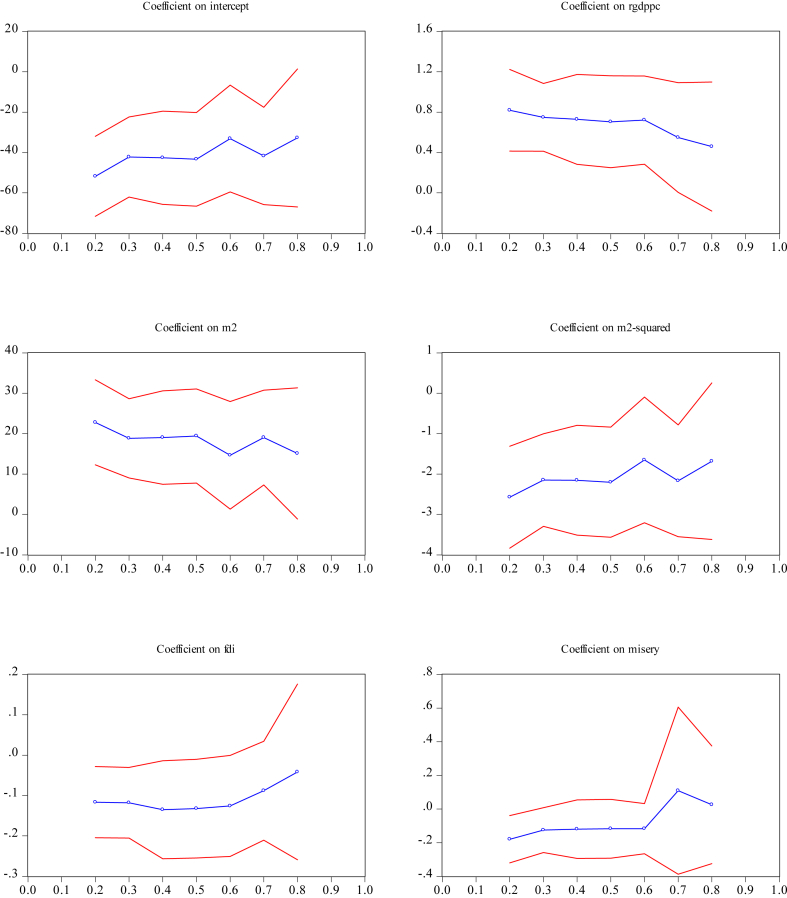
Quantile estimates for the shadow economy (proxy by M2/GDP ratio).

**Figure 5:**
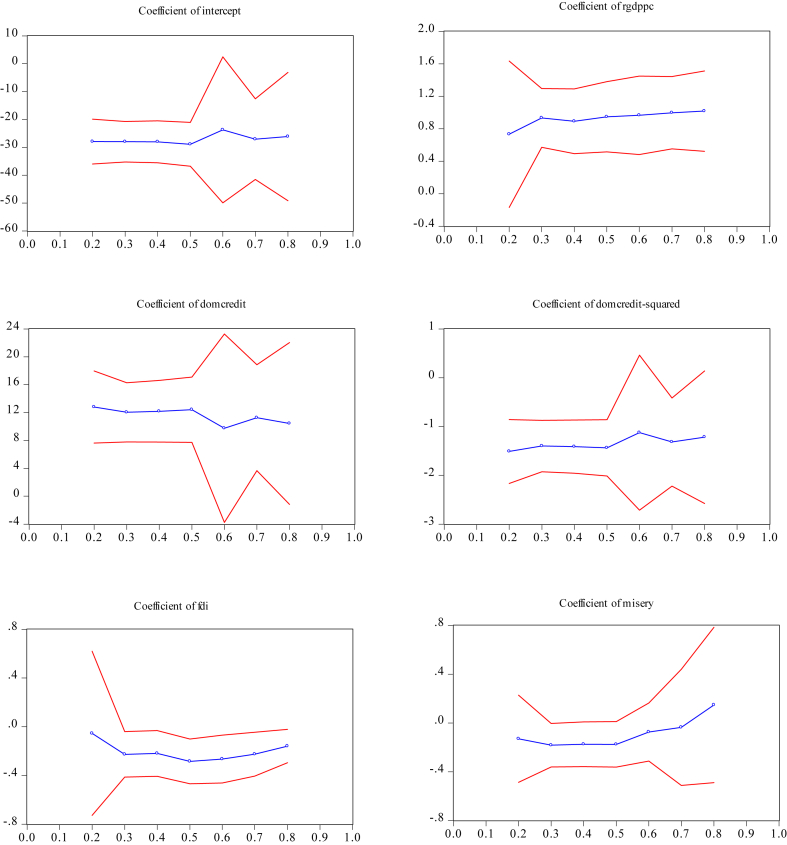
Quantile estimates for the shadow economy (proxy with domestic credit to GDP).

Figure 4 displays the graphical presentations of the quantile estimates for the ratio of M2 to GDP. Figure 5 exhibits the ratio of domestic credit to the private sector to GDP. The quantile estimates in Appendix Figures 4 and 5 show that the slope estimates at different quantiles show nonlinear patterns. These results suggest the existence of parameter heterogeneity across quantiles.

Table 6 shows the Wald test results on symmetry and slope equality. According to Koenker and Basset (1982), the slope equality test is also a robust heteroscedasticity test. The values of the Wald slope equality test equal to 26.68 (with *p*-values equal to 0.64) for M2 and 16.35 (0.979) for domestic credit. It implies that the coefficient estimates are not significantly different across the quantiles and have similar conditional quantiles. The Wald test of quantile symmetry gives 11 39 for M2 (with *p*-values equal to 0.876) and 8.75 (0.965) for domestic credit. These results indicate that the coefficients across all quantiles do not deviate significantly from symmetry.

Panel A and Panel B in Table 6 show that most variables are in the quantile regressions are significant at *α* = 1% (except for the misery index), although the coefficient signs are not always in line with our expectations because they vary with quantiles. These results provide support the claim that financial sector development can eradicate Indonesia's shadow economy.

The estimated coefficients for the financial sector development indicators in both panels demonstrate a quadratic and inverted U-shape curve with ${\hat{\text{θ}}}_{2}>0$ and ${\hat{\text{θ}}}_{3}<0$. Thus, the quantile regression provides evidence of the non-linear relationship between financial sector development and the shadow economy at all quantiles. The turning points where the size shadow economy starts to decline when the financial sector development expands lie between 4.39 and 4.49 in Panel A, and between 4.23 and 4.33 in Panel B. The turning points obtained from quantile regression are generally greater than those from the OLS in Panel A but smaller in Panel B.

FDI is another factor that curbs Indonesia's shadow economy. Results in both Panel A and Panel B show that the FDI variable is statistically significant *α* = 1% in most quantiles, but with a diminishing impact on curtailing the shadow economy. The coefficient estimates are higher at the lower quartiles but smaller at higher quantiles. For example, Panel A shows that at the 30^th^ quantile, a 10% increase in FDI reduces Indonesia's shadow economy by 1.2%; but at the 70^th^ quantile, a 10% increment in FDI reduces the shadow economy by 0.9%. Similar findings are shown in Panel B.

Table 6 shows that real GDP per capita increases the size of Indonesia's shadow economy at all quantiles of income. The impact of real GDP per capita on the shadow economy is larger at the lower quantiles compare to at the higher quantiles (see Panel A). For instance, at 20^th^ quantile, a 10% increase in real GDP per capita increases the shadow economy by 8.2%; at the 80th quantile, it increases the shadow economy only by 4.5%. Panel B also shows similar results. Generally, the misery index shows no impact on the shadow economy at 5% level of significance for all quantiles.

These results suggest that Indonesia's economic growth does not guarantee the same economic opportunity for the entire population. Nevertheless, we would expect that economic shocks, such as rising unemployment and inflation, still compel some people or firms to enter the shadow economy.

## 6 Conclusion

We estimate the size of Indonesia's shadow economy from 1980 to 2015. We develop a model to investigate factors that affected Indonesia's shadow economy, that is, income, financial sector development, foreign direct investment, and the misery index. We use the OLS, ARDL, FMOLS, DOLS, and CCR methods to estimate our long-run model. We also run the quantile regression on the model to have a more comprehensive analysis.

We find a non-linear relationship between financial sector development and Indonesia's shadow economy. This relationship demonstrates an inverted U-shape curve. The size of Indonesia's shadow economy expands at the earlier stages of financial sector development until a turning point and decreases when financial sector development enhances further. Our findings support the findings from previous studies, such as Bose et al. (2012). We also find that foreign direct investment can help to narrow the shadow economy. Meanwhile, increases in income expand the shadow economy; while the misery index show ambiguous impact on shadow economy in Indonesia.

Based on our findings, we suggest the Indonesian authorities focus on financial inclusion programs and financial sector reforms. For example, Bank Indonesia (Indonesia's central bank) and Otoritas Jasa Keuangan (Indonesia's Financial Service Authority) can issue regulations that widen access for the micro, small, and medium firms to the credit markets and promote reforms to the capital market. On the fiscal side, the Indonesian government should enhance existing programs to reduce poverty and narrow the income gap in the country. Fiscal policies and incentives that can attract more foreign direct investment into the country should also be encouraged.

## Declarations

### Author contribution statement

Sugiharso Safuan: Performed the experiments; Analyzed and interpreted the data; Wrote the paper.

Muzafar Shah Habibullah: Conceived and designed the experiments; Analyzed and interpreted the data; Wrote the paper.

Eric Alexander Sugandi: Analyzed and interpreted the data; Contributed reagents, materials, analysis tools or data; Wrote the paper.

### Funding statement

This work was supported by the Research Grant NKB-1912/UN2.R3.1/HKP.05.00/2019 from the Hibah Kolaborasi Riset Internasional 2019, 10.13039/501100006378Universitas Indonesia.

### Data availability statement

Data included in article/supplementary material/referenced in article.

### Declaration of interests statement

The authors declare no conflict of interest.

### Additional information

No additional information is available for this paper.
